# P-1081. Long-term Follow-up After Successful Discontinuation of Isolation for Carriers of Carbapenemase Producing Enterobacterales

**DOI:** 10.1093/ofid/ofaf695.1276

**Published:** 2026-01-11

**Authors:** Shadi Zahran, Daniel Grupel, Ilana Gross, Ayelet Favor, Naama Ronen, Tal Bendahan, Eleonora Radvogin, Nehama Shilo, Jana Hen, Miriam Ottolenghi, Yonatan Oster

**Affiliations:** Department of Clinical Microbiology and Infectious Diseases, Hadassah Medical Center, Jerusalem, Israel and Faculty of Medicine, Hebrew University, JerusalemIsrael, Jerusalem, Yerushalayim, Israel; Hadassah Hebrew University Medical Center, Jerusalem, Yerushalayim, Israel; Hadassah Hebrew University Medical Center, Jerusalem, Yerushalayim, Israel; Hadassah Hebrew University Medical Center, Jerusalem, Yerushalayim, Israel; Hadassah Hebrew University Medical Center, Jerusalem, Yerushalayim, Israel; Hadassah Hebrew University Medical Center, Jerusalem, Yerushalayim, Israel; Hadassah Hebrew University Medical Center, Jerusalem, Yerushalayim, Israel; Hadassah Hebrew University Medical Center, Jerusalem, Yerushalayim, Israel; Hadassah Hebrew University Medical Center, Jerusalem, Yerushalayim, Israel; Hadassah Hebrew University Medical Center, Jerusalem, Yerushalayim, Israel; Hadassah Hebrew University Medical Center, Jerusalem, Yerushalayim, Israel

## Abstract

**Background:**

The isolation of Carbapenemase producing Enterobacterales (CPE) carriers poses significant medical, psychosocial, economic and logistical burdens on patients and health systems. Hadassah Medical Center (HMC) follows national regulations for Discontinuation of Isolation (Dol) for CPE carriers. DoI is achieved by three consecutive negative tests for rectal CPE carriage, with a minimum of one week between the first and last. The first two are sent for cultures and the third for a PCR test. Data regarding the long-term consequences following successful Dol is lacking.

**Figure:**
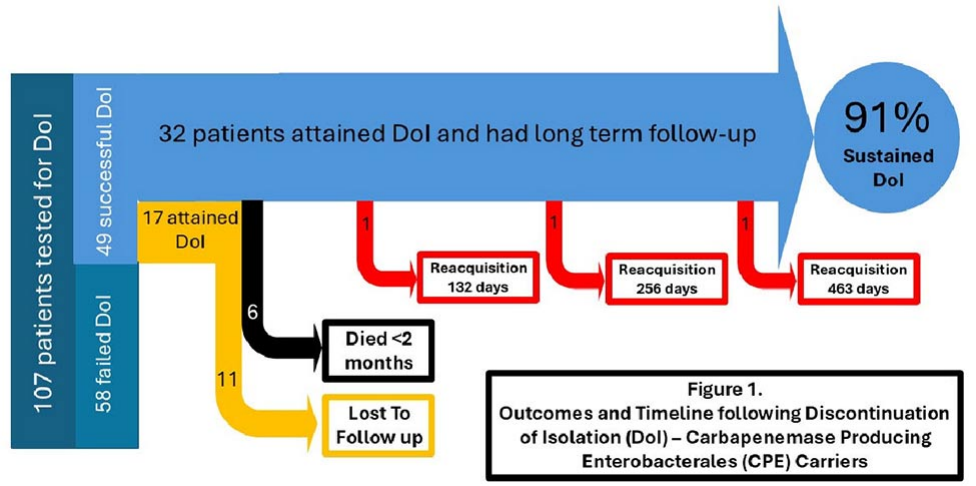

**Figure:**
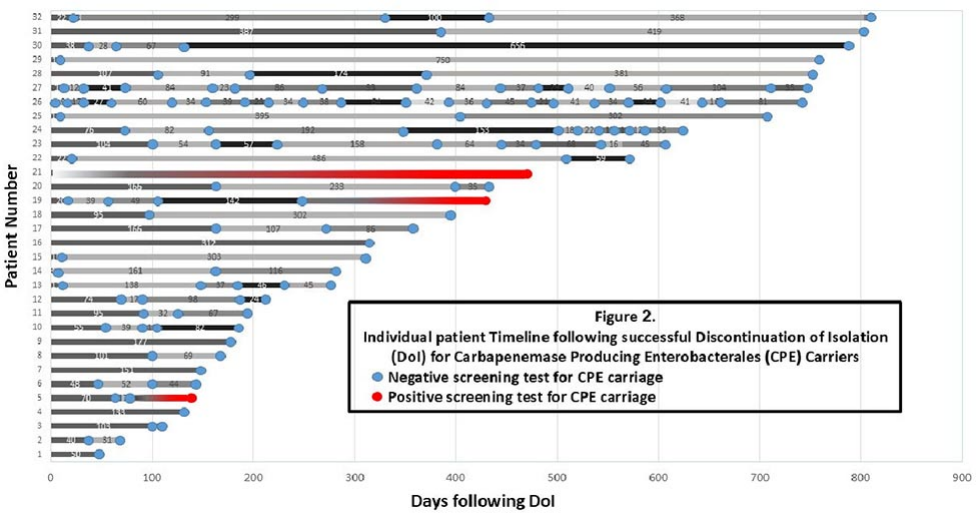

**Methods:**

We retrospectively evaluated 107 Patients undergoing a DoI attempt from July 2022 to February 2023. We reviewed baseline characteristics, including age, sex, comorbidities, residence, hospitalization, antibiotic treatment in the previous three months, CPE carriage details, and duration successful DoI. Outcomes assessed were time to reacquisition of CPE and death.

**Figure:**
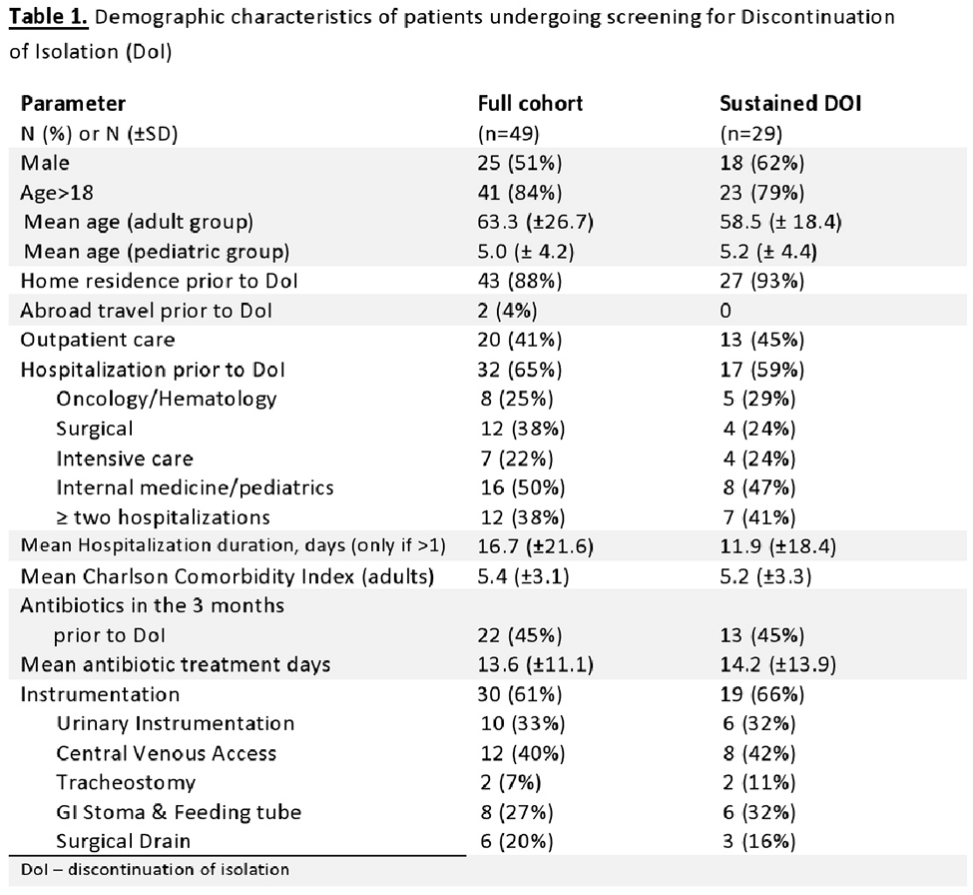

**Figure:**
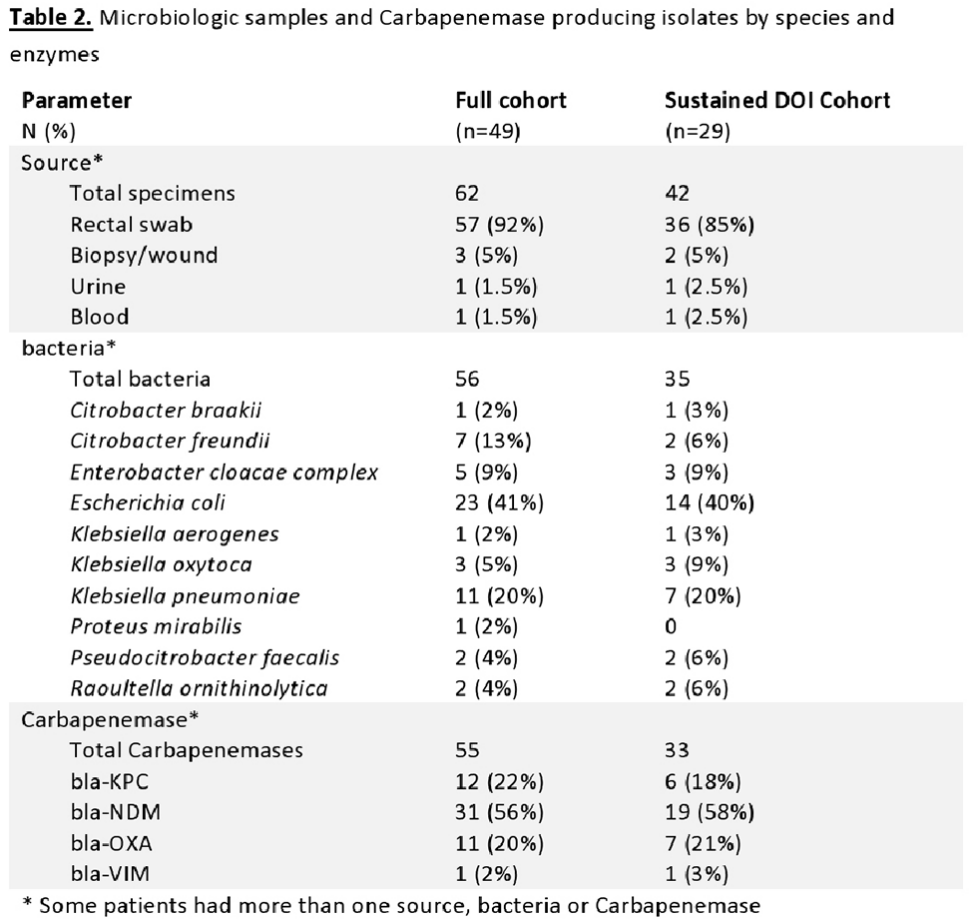

**Results:**

We identified 49 patients (46%) with successful DOI, 51% of whom were male. The mean age of the adult cohort was 63 years (SD ±17.2). Twenty-four Patients (49%) had an active malignancy, and the median Charlson comorbidity index for the cohort was 5.4.

Hospitalizations prior to DoI, outpatient visits, antibiotic treatment, instrumentation, and medical procedures were common (Table 1). The prominent microbiologic isolate was E. coli (38%), and the leading Carbapenemase gene identified was bla-NDM (Table 2).

Of the 49 patients attaining DoI, 32 (64%) were retested with a mean of 4.4 tests each. (Figure 1) Twenty-nine of them (91%) remained negative. The average time of proven sustained DoI was 415 days (IQR 492 – 683) (Figure 2); four died during the follow-up period. Only three patients (9%) were found to reacquire CPE carriage 132, 256, and 463 days following DoI.

Seventeen patients (34%) were never retested; five of those died during the follow-up period.

**Conclusion:**

Sustaining prolonged CPE DoI is feasible even in complex and debilitated patients, as 91% of our cohort remained CPE-free over a two-year follow-up. Requisition of CPE carriage is rare, and the unlikely risk of occult CPE carriage and transmission should not deter prevention teams from applying DoI protocols.

**Disclosures:**

All Authors: No reported disclosures

